# Long-Term Antibody Response and Vaccination Efficacy in Patients with COVID-19: A Single Center One-Year Prospective Study from the Czech Republic

**DOI:** 10.3390/v14030526

**Published:** 2022-03-04

**Authors:** Miroslav Fajfr, Radek Sleha, Sylva Janovska, Vladimir Koblizek, Mikulas Skala, Stanislav Plisek, Petr Prasil, Petr Smahel, Pavel Bostik

**Affiliations:** 1 Institute of Clinical Microbiology, University Hospital, 50005 Hradec Kralove, Czech Republic; fajfrm@lfhk.cuni.cz; 2 Faculty of Medicine in Hradec Kralove, Charles University, 50003 Hradec Kralove, Czech Republic; koblizekv@lfhk.cuni.cz (V.K.); skalamik@lfhk.cuni.cz (M.S.); pliseks@lfhk.cuni.cz (S.P.); prasilp@lfhk.cuni.cz (P.P.); smahelp@lfhk.cuni.cz (P.S.); 3 Department of Epidemiology, Faculty of Military Health Sciences, University of Defence, 50001 Hradec Kralove, Czech Republic; radek.sleha@unob.cz (R.S.); sylva.janovska@unob.cz (S.J.); 4 Department of Pneumology, University Hospital, 50005 Hradec Kralove, Czech Republic; 5 Department of Infectious Diseases, University Hospital, 50005 Hradec Kralove, Czech Republic

**Keywords:** serology, SARS-CoV-2, virus neutralization test, ELISA, dynamics, vaccination, reinfection

## Abstract

Background: The diagnosis of SARS-CoV-2 is almost exclusively performed by PCR or antigen detection. The detection of specific antibodies has not yet been considered in official diagnostic guidelines as major laboratory evidence for a case definition. The aim the present study is to analyze antibody responses in outpatient and inpatient cohorts of COVID-19 patients in the Czech Republic over a 12-month period, and assess the potential of antibodies as a diagnostic tool. Methods: A total of 644 patients was enrolled in the prospective study. IgA, IgM and IgG antibody levels, as well as virus neutralization titers, were analyzed over a 12-month period. Results: Our study showed low antibody positivity levels at the admission. However, at 2 weeks after infection, 98.75% and 95.00% of hospitalized patients were IgA and IgG positive, respectively. Even in the outpatient cohort characterized by milder disease courses, the IgG antibody response was still sustained at 9 and 12 months. The data show a high correlation between the IgG levels and virus neutralization titers (VNTs). Samples from later time-points showed positive antibody responses after vaccination in both cohorts characterized by high IgG levels and VNT over 1:640. The samples from unvaccinated persons indicated a relatively high level of reinfection at 6.87%. Conclusions: Our results show that the detection of antibodies against the SARS-CoV-2 shows an increasing sensitivity from week 2 after infection and remains highly positive over the 12-month period. The levels of IgG antibodies correlate significantly with the VNTs. This suggests that the serological data may be a valuable tool in the diagnosis of SARS-CoV-2 infection.

## 1. Introduction

Human coronaviruses represent well-known pathogens of respiratory tract infections ranging in severity from a seasonal common cold to severe respiratory distress syndromes. In 2019, the seventh pathogenic human coronavirus was first described in Wuhan Province, China, and from the beginning of 2020 the global pandemic of coronavirus disease named COVID-19 caused by this new coronavirus developed. The virus named SARS-CoV-2 subsequently caused infections in more than 172 million people, which led to more than 3.7 million deceased patients all over the world. The Czech Republic became one of the most heavily affected countries with 282 deaths per 100,000 inhabitants. The country ranked third worldwide and second in the EU, according to the relative number of deaths [1]. Several criteria have been used for the diagnosis of COVID-19. A suspected case definition of this disease was characterized by a combination of clinical and epidemiological criteria both in the Center for Disease Control and Prevention (CDC) and World Health Organization (WHO) guidelines. The laboratory confirmation was made by the detection of SARS-CoV-2 nucleic acid using PCR or the positivity of SARS-CoV-2-specific antigen according to the WHO guidelines. The CDC guidelines, however, also accept a supportive role of the serological detection of specific antibodies for the diagnosis of SARS-CoV-2 infection [2,3].

Nucleic acid amplification tests (NAATs) are characterized by a high cumulative sensitivity (over 98%) and allowed for the diagnosis several days before the onset of symptoms [4,5]. The antigen detection tests showed a very variable sensitivity, with cumulative numbers only at 56.2% [4]. The serology of SARS-CoV-2 had its limitations in the absence of data from larger studies, which were the reason for classifying the serology as a supportive diagnostic tool only. According to some studies, there were significant differences between the results for the sensitivity and specificity, depending on several variables. For example, the tests showed variations related to differences in the study designs in comparative studies. Thus, some studies showed a higher cumulative sensitivity of chemiluminescent microparticle assay (CLIA) tests (97.5%) compared to enzyme-linked immunoassay (ELISA) tests (90.7%) [6,7], but other reports showed opposite trends [8,9]. In addition, some data indicated a relatively high sensitivity of ELISA tests, detecting either anti-RBD or anti-N specific antibodies [7,8,9]. Yet, another study suggested a better sensitivity of ELISA tests detecting anti-N antibodies during the early stages of infection [10]. The serological diagnostic methods showed a relatively low sensitivity during the first 7–10 days of infection (average sensitivity around 30%) according to some reports, but the sensitivity rapidly increased from week 2 of infection up to over 95% between days 22–35 [6,11]. There has been a relative lack of studies focusing on antibody responses over longer periods of time, but a recent study assessed anti-spike protein-specific IgG antibodies over the period of 6 months [12]. Thus, the aims of this study are (a) to provide a long-term analysis of antibody responses in large COVID-19 in- and outpatient cohorts from the Central European region for the first time; (b) provide support for the notion that specific antibodies can be used as proof of the ongoing or passed disease, specifically for the situations in which a direct identification of the virus is negative; (c) show a correlation between VNTs and specific antibody levels, which can then be used as predictors of the antibody neutralization capacity; and (d) confirm the efficacy of the vaccination. Therefore, the data reported herein show the results of an analysis of the dynamics of specific antibody responses in COVID-19 patients from the 12-month prospective study conducted at the University Hospital in Hradec Kralove, Czech Republic.

## 2. Materials and Methods

### 2.1. Participant Enrolment and Sample Collection

The enrolment of participants started during the first COVID-19 wave in the Czech Republic in April 2020, and is still ongoing. In this study, samples from a total of 566 patients were analyzed in 2 separate cohorts. Cohort A consisted of patients with a more severe course of the disease, who required hospitalization either in standard wards or in intensive care units. The outpatient cohort B contained patients with milder courses of disease, recruited from both the hospital and general practitioners in the area. All participants in this study were confirmed as SARS-CoV-2 positive by a PCR test from a nasopharyngeal swab. Serum samples from the patients enrolled in cohort A were collected at the following time points—at admission, at 2 weeks, 3 months, 6 months, 9 months and 1 year. Samples from the outpatients (cohort B) were obtained at 2 weeks, 3 months, 6 months, 9 months and 1 year after diagnosis. The numbers of samples collected at each time point vary due to the differences in the compliance of the patients. Each serum sample was divided into 4 aliquots, where 1 was used for antibody level analysis and the remaining 3 were frozen at −80 °C for further use. The study was approved by the University hospital in Hradec Kralove Ethical Committee (No. 202011186P) and informed consent was obtained from all participants.

The anamnestic data showed that 69.33% of patients from cohort A and 52.83% of patients from cohort B received vaccination during the covered time period of 12 months after infection. In both groups, a dominance of Comirnaty (BioNTech^®^/Pfizer^®^, Mainz, Germany) vaccine was determined; 75.82% in cohort A and 83.45% in cohort B. The individual patients were immunized at various times post-infection, starting between the visits at 3 and 6 months. Thus, high numbers of samples from patients after vaccination were collected at months 6, 9 and 12 (cohort A: 61.54–81.32%; cohort B: 26.61–62.59%).

### 2.2. Serum Antibody Determination

The aliquots of fresh serum samples were utilized for the analysis of the antibody levels directly after the sera were received in the laboratory at the Institute of Clinical Microbiology, University Hospital in Hradec Kralove. The semi-quantitative ELISA kits, EI SARS-CoV-2 IgG (Euroimmun, Lübeck, Germany), EI SARS-CoV-2 NCP IgM (Euroimmun) and EI SARS-CoV-2 IgA (Euroimmun) were used. The antigens utilized in these assays were as follows—nucleocapsid antigen for the IgM and S1 domain of S antigen for the IgA and IgG analyses. All ELISA tests were performed according to current SOPs and manufacturer specified procedures, using an ETIMAX device (DIASorin^®^, Saluggia, Italy). The results of the assays are expressed as index S/CO. The evaluation criteria were identical for all assays—negative results with an index < 0.8, borderline results with values between 0.8 and 1.1, and the positive results with an index ≥ 1.1.

### 2.3. Virus Neutralization Assay

The virus neutralization assays were performed in the BSL3 laboratory facility of the Faculty of Military Health Sciences, University of Defence in Hradec Kralove. The wild SARS-CoV-2 origin variant strain (provided by Professor Ruzek, University of South Bohemia) and SARS-CoV-2 Alpha variant strain (provided by Doctor Jirincova, National Institute of Public Health) were propagated on a VERO CCL81 cell line, which was also used for the determination of neutralization antibody titers. The virus neutralization assay was performed, as previously described by Manenti et al. [13]. Briefly, the serum samples were heat-inactivated for 30 min at 56 °C. Serial 2-fold dilutions (starting at 1:20) of patient serum samples in Dulbecco‘s modified Eagle’s medium (DMEM) were then prepared in 96-well plates. Subsequently, each dilution was mixed with an equal volume of virus solution of a concentration 2000 tissue culture infectious dose 50 (TCID_50_)/mL). The serum–virus mixture was incubated for 1 h at 37 °C in a humidified atmosphere with 5% CO_2_. After the incubation period, 100 µL of these mixture samples at each dilution was added in duplicates to a 96-well plate containing a semi-confluent Vero cell monolayer. The plates were incubated at 37 °C in a humidified atmosphere with 5% CO_2_. After 3 days, the plates were evaluated using an inverted microscope. The virus neutralizing antibody titer (VNT) was determined as the highest serum dilution that prevented the development of the cytopathic effect (CPE) in duplicate wells.

### 2.4. Statistical Evaluation

The GraphPad Prism 9 software (version 9.20, GraphPad Software Inc., San Diego, CA, USA) was utilized for graphical outputs and basic statistical evaluation. The normality evaluation was performed using the Anderson–Darling test and Shapiro–Wilk test. Normally, the distributed data are analyzed using a one-way ANOVA with post hoc Student *t*-test. Non-normally distributed data were analyzed by the Kruskal–Wallis test with post hoc Mann–Whitney test or Dunn’s multiple comparisons test. The differences were considered significant when *p* ≤ 0.05. A statistical evaluation of the correlations between the antibody levels obtained by ELISA and VNT was performed using the Spearman’s rank correlation coefficient.

## 3. Results

### 3.1. Demographic Data

Samples from a total of 644 patients divided into 2 cohorts were analyzed in the study (Table 1). Cohort A (those requiring hospitalization) consisted of 225 patients, where 38.67% of the patients was female with the median age of 70 years, and 61.33% was male with the median age of 69 years. The second cohort, B, consisted of patients with milder courses of infection allowing for the outpatient treatment. In his cohort, out of the 419 patients, 31.99% was female (median age: 41 years) and 68.01% was male (median age: 42 years).

### 3.2. Serum Antibody Levels

**Figure 1:**
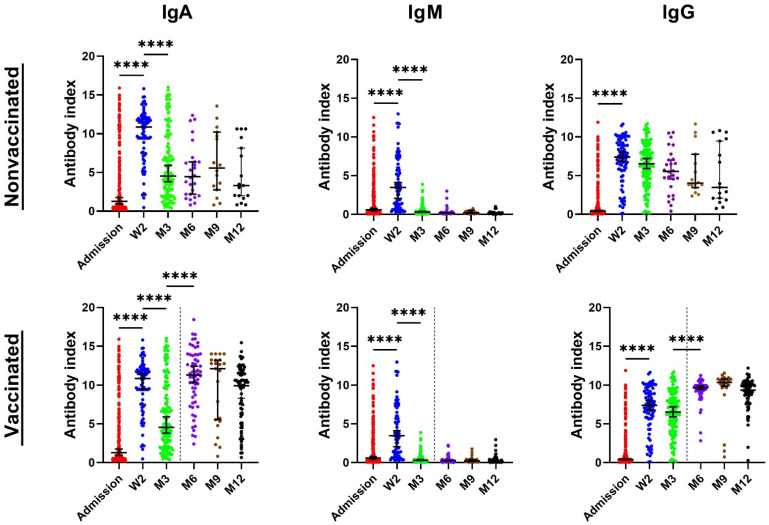
The detected antibody levels in the sera of cohort A over the 12-month period expressed as index values. Data are presented as the median ± 95% confidence intervals. The dotted line indicates the time when some patients started to be vaccinated. The nonparametric ANOVA Kruskal–Wallis post hoc Dunn’s multiple comparisons test was used for statistical evaluation. **** represent statistical significance *p* < 0.0001. The absence of * marks means no significance.

The sera from patients in cohort A for the antibody analyses were collected at admission, then at 2 weeks, and 3, 6, 9 and 12 months (Figure 1, Supplementary Table S1). 

At the time of admission, IgA antibodies were the most prevalent class and reached the highest index values. Their levels were positive in 52.17% of patients with the index value average of 6.52 (range: 1.103–15.905). The second most prevalent IgM antibodies were detected in 37.20% of patients with the index value average of 4.38 (range: 1.141–12.513). As expected, the IgG class showed the lowest results, being positive in 28.99% of patients with the index value average of 4.93 (range: 1.174–11.893). At week 2, after symptom onset, all antibody classes showed a rapid increase in both the positivity and index values. Nevertheless, however, the IgA class exhibited the highest positivity prevalence among patients (98.75%) with the index value average of 9.98. The antibodies in the IgM and IgG classes showed positivity levels in 77.75% and 95.00% of patients, respectively. At month 3, the IgA and IgG remained highly prevalent and positive in 90.52% and 94.83% of patients, respectively. As expected, the IgM positivity decreased at that point, but remained detectable in 14.66% of patients. Additionally, very similar results were obtained at month 6, with sufficient positivity in IgG (98.81%) and IgA (97.62%). Samples from the time periods of 9 months and 1 year post infection showed a positivity of IgG in 97.44% and 96.70% of patients.

**Figure 2:**
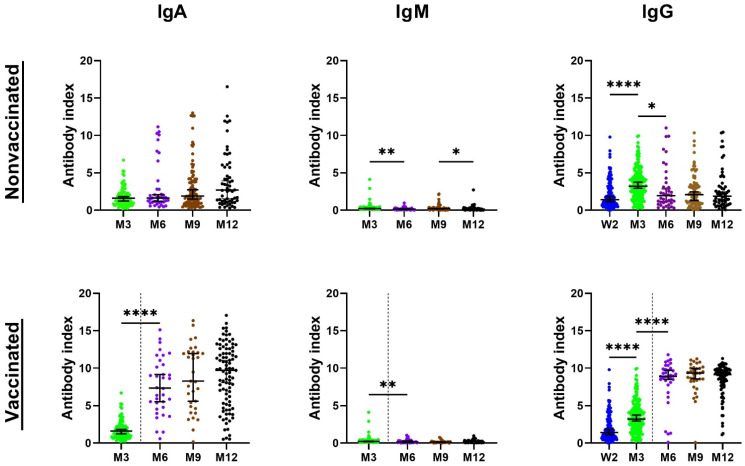
The detected antibody levels in the sera of cohort B over the 12-month period expressed as index values. Data are presented as the median ± 95% confidence intervals. The dotted line indicates the time when some patients started to be vaccinated. The nonparametric ANOVA Kruskal–Wallis post hoc Dunn’s multiple comparisons test was used for the statistical evaluation. Statistical significance is labeled as follows: **** *p* < 0.0001, ** *p* < 0.0075; * *p* < 0.0424.

The character of cohort B did not allow for obtaining the “time 0” samples corresponding to the admission samples in hospitalized patients, but the follow-up samples were obtained at time periods corresponding to cohort A—months 3, 6, 9 and 12. In general, however, the antibody response among the outpatients was slower, and the numbers of patients showing positive antibodies and the antibody levels in the positive patients (index values) were lower than those found in cohort A (Figure 2). 

Thus, at week 2, only 59.12% of patients were positive for IgG with the index value average of 3.09. At 3 months post infection, the positivity numbers were highest in the IgG class (86.84%) followed by the IgA (69.16%). IgM class antibodies were the least prevalent and found in only 2.80% of patients. The IgG and IgA classes were still highly prevalent at months 6 and 9 post symptom onset, but, interestingly, with “switched” positions. Thus, 82.19% and 86.30% of patients tested positive for IgG and IgA, respectively, at 6 months. Similarly, at month 9, IgG and IgA antibodies were positive in 76.61% and 78.26% of patients, respectively. At a 1-year time point, out of 147 patients, 88.44% were positive for IgA and IgG. 

At any time point during the follow-up, the average and median levels of positivity were higher in cohort A than in cohort B in the IgG and IgA antibody classes. In addition, the average and median levels of positivity of IgA antibodies were always higher than the IgG levels at corresponding time points. The statistical evaluation of the results was preceded by a normality test and all evaluated collection times passed the normality test on Alpha = 0.05 level. The subsequent statistical analysis (Supplementary Table S1) found significant differences in antibody positivity between cohorts A and B in antibodies of all classes at week 2, month 3, month 6, month 9 and month 12 (*p* < 0.05), with the exception of IgM at month 9 (*p* = 0.2152). At month 12, the differences were statistically significant only in the IgG class; antibody levels in IgM and IgA classes showed non-significant differences (*p* = 0.1098 and *p* = 0.095, respectively).

The serological results from months 6, 9 and 12 were affected by reinfections and by the fact that increasing numbers of patients of both cohorts had been vaccinated over time. As a result, detectable increases in the median and average levels of antibodies were detected in these patients. The reinfection was defined as at least a four-fold increase in the virus neutralization titers in samples from patients without a vaccination history. Thus, a total of 4.08% of patients showed an increase in antibodies at month 3. However, at this time point, these patients are more likely to be late antibody producers after the primoinfection. At month 6, a total of 7.69% of patients from cohort A without vaccination and 2.56% from cohort B fit the definition of reinfection. In the non-vaccinated patients from cohort B, serological markers of reinfection were found in 7.69% and 9.09% of patients at months 9 and 12, respectively. In cohort A, the reinfection was found only at month 12 in 5.88% of patients. The overall mean reinfection level in all non-vaccinated patients at all time points was calculated as 6.87% (data not shown). None of these patients had severe reinfection requiring hospitalization. On the contrary, the majority of reinfections was asymptomatic, showing a good protection against more severe courses of disease after primoinfection. Interestingly, among hospitalized patients, a total of 1.4% and 9.4% of patients did not show any detectable antibody response during the first 3 months in cohorts A and B, respectively. During the entire study period, 27 patients from cohort A died. However, contrary to the results published by Youngche et al. [14], there was no detectable increase in antibody levels in these patients compared to the surviving individuals. 

**Figure 3:**
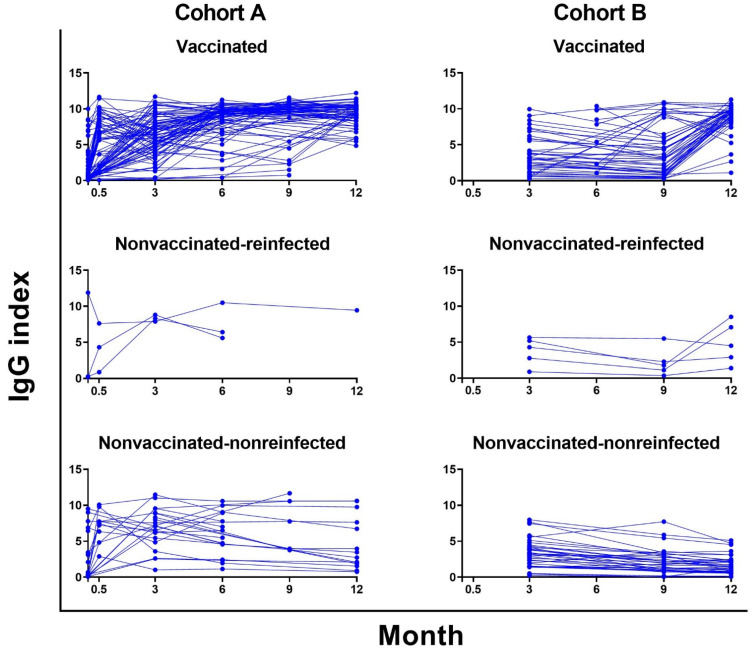
The dynamics of IgG antibodies in individual patients and the effect of the vaccination and reinfection in patient cohorts A (left column) and B (right column). The charts depict significant increases in antibody levels in groups of vaccinated and non-vaccinated reinfected patients at month 12.

Further analysis of the levels of specific antibody responses in consecutive samples from individual patients over time showed additional interesting results (Figure 3).

Thus, in cohort A, the levels of IgG exhibited the characteristic dynamics with a rapid increase in week 2, followed by a significant decrease in these values. On the contrary, the dynamics of IgG in cohort B showed a much flatter course, where only relatively small differences in the levels of the consecutive samples were observed. The results also show slow waning in non-vaccinated patients without reinfection in both cohorts overall, where only a very small number of individuals developed rapid antibody waning. The antibody dynamics in both vaccinated patients and non-vaccinated patients with reinfection showed the expected increases of IgG levels in both cohorts (Figure 3).

Because of a high number of vaccinated patients in months 6, 9 and 12 (cohort A 61.54–81.32%; cohort B 26.61–62.59%), the antibody levels were skewed due to an increase after vaccination. Therefore, the data were re-analyzed according to the vaccination history in two categories—patients vaccinated with the second dose less than 4 months before sampling and those vaccinated more than 5 months before (Supplementary Table S2a,b). In the samples from patients without or before vaccination, decreases in IgG positivity between months 6 and 12 in both cohort A (from 95.83% to 87.50%) and cohort B (from 71.05% to 66.00%) were detected. The average and median positivity also decreased, but a higher positivity percentage and average/median were always detected in the samples from cohort A compared to cohort B. Subsequently, a comparison of antibody levels between the samples from patients vaccinated in less than 4 months and more than 5 months was performed by the nonparametric Mann–Whitney test. It showed significantly higher values in the IgG antibody class in both cohorts (*p* = 0.0042 to 0.0399), in samples from patients vaccinated up to 4 months before sampling (statistical data available in the Supplementary Table S2b). Summary of the data from the reinfected patients is available in Supplementary Table S2c.

### 3.3. Virus Neutralization Titers

**Figure 4:**
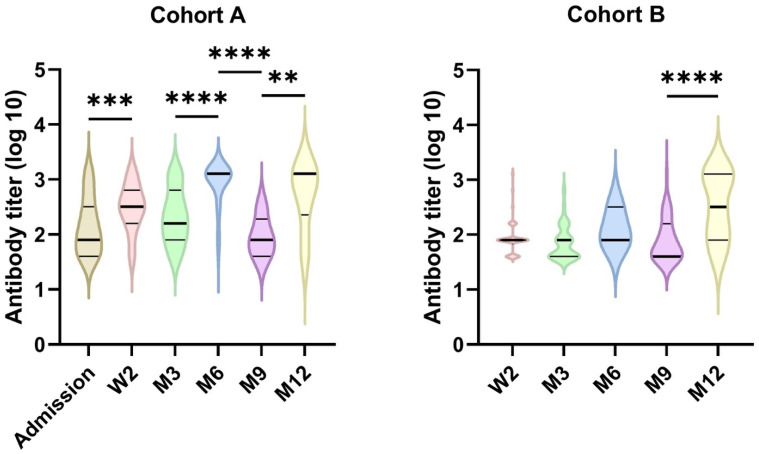
The dynamics of virus neutralization titers in time. The graph illustrates the higher titers in the patients from cohort A in general, and increases in the titers between months 9 and 12 in both cohorts due to vaccination. The statistical evaluation was performed using the nonparametric Mann–Whitney test with the significance set at *p* < 0.05. **** denotes the statistical significance at *p* < 0.0001, *** at *p* = 0.0002 and ** at *p* = 0.0036.

Virus neutralization antibody titers (VNTs) represent a critical parameter of antibody response, indicating a real capacity for antibodies to neutralize the virus. To investigate virus neutralization potential of the sera from our patients, all of the collected samples were tested for their neutralization capacity, in parallel with the Ig levels, using the original variant of live SARS-CoV-2 virus. The results show that, in cohort A, 56.10% of the samples showed VNTs over 1:40 and only 21.46% generated neutralization titers of 1:160 and higher at the time of admission. At the subsequent timepoints of 2 weeks and 3 months, the titers of 1:160 and higher were detected in 84.15% and 63.79% of patients in cohort A, respectively, where the increase in VNTs at the 2-weeks timepoint was highly significant (Figure 4). In the patients from cohort B, the corresponding patient positivity was 41.54% and 16.73%, respectively. Then, a rapid decrease in the numbers of non-vaccinated patients from months 6, 9 and 12 was observed, showing the titers of 1:160 and higher only in 64.29% to 33.33% in cohort A, and 30.00% to 27.67% in cohort B. The levels of the VNTs in cohort B were generally lower than in cohort A (Figure 4).

Because of the increasing numbers of samples from vaccinated patients at 6, 9 and 12 months, the overall VNT levels increased and the 1:160 and higher titers were found in 88.68 to 78.58% of the samples in cohort A and 33.33 to 50.91% in cohort B (see Supplementary Table S3). Thus, in both cohorts, statistically significant (*p* < 0.05) increases in the VNT levels were found, specifically at month 12. Interestingly, a highly significant increase in VNT levels was also found at month 6. In patients from both cohorts vaccinated within a period of 4 months before sampling, increased levels of antibodies correlated to higher VNTs (over 1:640), with only few exceptions in both cohorts. 

**Figure 5:**
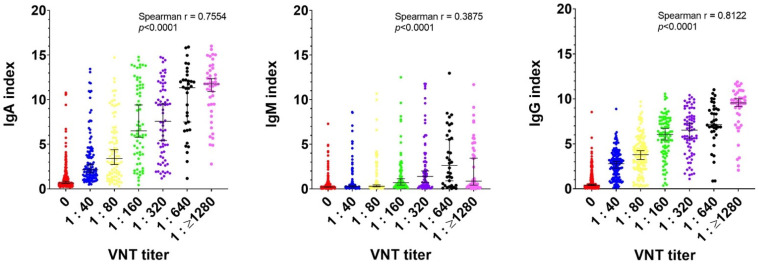
Correlation of the antibody levels and virus neutralization test. The Spearman’s rank correlation coefficient test shows a positive correlation of specific IgG antibody levels and VNT.

The statistical evaluation of correlations of the levels of all three antibody classes and the respective VNTs in cohort A and cohort B individually, and their subsequent comparison between the cohorts, showed no significant differences (data not shown). Therefore, this permitted the calculation of the correlation of the VNTs and the Ig levels in all samples together, regardless of the mode of treatment (inpatient or outpatient). Figure 5 shows the correlations between the IgG, IgA and IgM levels and the VNTs in the individual serum samples. The results show a strong non-linear positive correlation (Spearman’s r = 0.8122; *p* < 0.0001), only in the IgG class. The IgA antibody levels showed only a moderate correlation (Spearman’s r = 0.7554, *p* < 0.001), while the IgM antibody levels showed a negative correlation (Spearman’s r = 0.3875, *p* < 0.001). 

**Figure 6:**
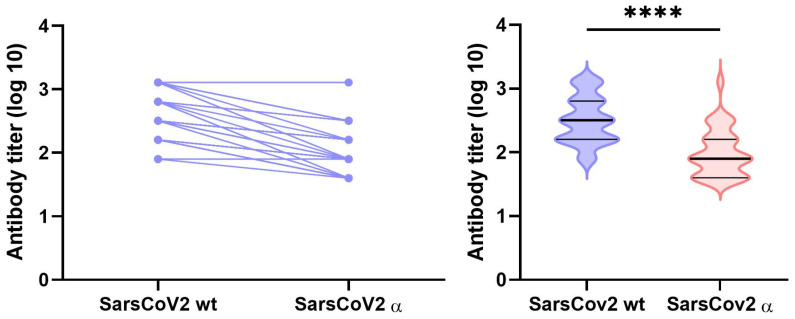
Neutralization of the SARS-CoV-2 Alpha variant with serum samples from patients infected with the original virus variant. The left panel shows a correlation of the virus neutralization titers against the original variant and the Alpha variant by the individual sera. The right panel shows the cumulative values of this analysis. In both panels, decreases in the VNT levels against the Alpha variant are observed. The nonparametric Mann–Whitney test was used for the analysis and **** represents the significance at *p* < 0.0001.

Subsequently, a comparison of the virus neutralization properties of antibodies from patients after infection with the original variant of SARS-CoV-2 to their neutralization efficacy against the Alpha variant of the virus was performed (Figure 6).

For this analysis, 75 samples were selected. The samples were chosen from both cohorts, where 40 and 35 samples were from cohorts A and B, respectively. Out of these samples, 45 samples showed VNT positivity (both low titer and high titer) and 30 showed VNT negativity against the original virus variant. Almost all VNT-negative samples remained negative with the Alpha variant, except for one showing a low neutralization titer of 1:40 (data not shown). Out of the 45 positive samples, only 2 (4.4%) exhibited titers against the Alpha variant, identical to those detected with the original virus, while 43 samples (95.6%) showed a decrease in the neutralization titers. A total of 11 samples showed decreases of 1 titer level and 32 samples showed decreases of 2–5 titer levels, compared to those obtained with the original variant. These data thus confirm a lower neutralization efficiency of the antibodies in potential reinfection with a different variant of the virus (Figure 6).

## 4. Discussion

At the beginning of the SARS-CoV-2 pandemic, the analysis of specific antibodies had a minor role in the disease diagnosis compared to the rapid antigen tests or NAAT tests. The reason was a lack of knowledge about the antibody level dynamics. However, with the increasing body of information about interactions of SARS-CoV-2 with the immune system, several studies in the field of COVID-19 serology indicated that it may become a valid diagnostic tool [6,7,8,10,12,14,15]. Several publications comparing different analytical methods—both EIA and CMIA—have shown slightly different results depending on the study design [8,16,17,18,19]. According to these studies, the EUROIMMUN assays used in our study have been among those showing the highest sensitivity and the closest correlation to the VNT results. The review by Gao et al. identified many risks or predictor factors associated with a severe course of COVID-19 disease [20]. These included the male sex (with underlying differences in hormone levels or expressions of ACE2 receptors) and older age (connected to an increased frequency of comorbidities or weakening of immunity). Similar demographic differences were found in our study, manifesting as a higher prevalence of older patients in cohort A, and male dominance in both cohorts. 

Despite the fact that some patients tested positive for IgA and/or IgM antibodies after day 5 of infection, the sensitivity of serological tests was relatively low during the first week after infection. Similarly to other studies, a higher sensitivity was observed during the 2nd and 3rd weeks of infection [4,5,10,21]. Thus, immediately after admission (up to 7 days after infection), IgA was positive only in 52% and IgG in 28.99% of all PCR-positive patients from cohort A. In the samples collected during the period from 14 to 21 days after admission to the hospital, the positivity of serological tests rose to 98.75% and 95% in the IgA and IgG antibodies, respectively. Incidentally, these were also the peak positivity numbers in cohort A. However, the subsequent samples in this cohort still showed a very high positivity over 90% in the IgG and IgA antibody classes at months 3, 6, 9 and 12. 

Samples from cohort B showed a delay in the increase in specific antibodies, which peaked until month 3. Higher positivity values detected at months 9 and 12 were compromised by reinfection for some patients. These findings thus suggest delayed antibody responses in mild or asymptomatic cases. The higher average and median value levels of IgA compared to those of IgG at all times was an interesting finding previously described only by Fourati et al. [22]. This study has also shown a better correlation of the 28-day mortality with the IgA, rather than with the IgG antibody levels. Considered together, this and our data both indicate the importance of IgA levels as one of the predictive factors. 

Many studies reported a short-term evaluation of specific antibodies in COVID-19 patients, but only a limited number of studies reported serological results in these patients during longer periods [12,23,24]. Our long-term data, similar to these publications, suggested a much longer duration of the specific humoral immunity than we presumed at the beginning of the pandemic. Thus, our data showed a sustained high positivity of IgG antibodies in 87.50% of non-vaccinated patients from cohort A and in 66.00% of cohort B non-vaccinated patients at the 12-month visit. Similar to others, our data also showed that the values of antibody indices decrease over time, which was manifested by relatively low average index values ranging between 3.21 to 3.15 in the outpatient group at later time points, and slightly higher average index values of 5.45 to 5.38 in hospitalized patients. 

Our study showed a higher percentage of positive antibody results for all the antibody classes in all the samples for a group of patients needing hospitalization compared to the outpatient group. Additionally, the average and median values of antibody levels in all the positive samples were significantly higher in cohort A. The dynamics of SARS-CoV-2-specific antibodies in both our cohorts showed a mild antibody waning in the majority of patients, similar to Chia et al. [15]. However, in several cases in our cohort, a delayed antibody response was observed. Another interesting result described here relates to the different dynamic of antibody levels in cohorts A and B. Thus, only moderate differences in the average antibody values of IgG over the entire time course of the study were observed in the outpatients, which probably relates to the differences in the viral loads between both cohorts.

The evaluation of the virus neutralization capacity showed that 23.08% to 38.89% of the outpatient samples exhibited VNTs above 1:40 at week 2 and month 3, respectively. At the same time, however, high numbers of the inpatient samples, 84.15% and 62.73%, showed VNTs of 1:160 and higher at the corresponding time points. These findings of the differences in the VNTs between patients with severe and mild courses of COVID-19 disease correspond to the previously published data [23,25]. The correlation between antibody levels and VNTs, which was found in our study, is in accordance with previously published data [26,27,28]. Our data thus show that only the levels of IgG class antibodies exhibit a strong correlation to the serum virus neutralization capacity. This suggests that the IgG levels can be used as the prediction factor for the estimation of neutralization antibody levels. The advantage of estimating VNTs on the basis of the IgG levels can thus be used as an indicator of immune protection after SARS-CoV-2 infection or vaccination, as was suggested the Khoury et al. [26]. The reinfection rates by seasonal coronaviruses were observed in different studies with a high frequency of 28% in NL63 and 66% in 229E human coronavirus infections during the first year after infection [29]. In our study, the serological marks of reinfection were found in 6.87% of patients from both cohorts. These relatively high numbers of reinfection are probably related to the introduction of new of SARS-CoV-2 variants during the course of our study. Thus, at the beginning of year 2021, the new variant, B.1.1.7 (called the Alpha variant), became the dominant strain. Our data on a subgroup of patients indeed show a lower VNT capacity of sera from patients infected with the original strain against the Alpha variant.

The results also indicate that vaccination induces strong humoral immune responses. The vaccinated patients in both cohorts developed a strong enhancement in antibody levels after vaccination. All vaccinated patients showed notable IgG and IgA antibody increases compared to the non-vaccinated population. In addition, a majority of samples from vaccinated patients showed very high VNTs (over 1:640) over the following 3 months after vaccination. These data thus confirm a clear benefit of the vaccination in the population previously infected with COVID-19. The interesting findings of the IgA level increases after intramuscular vaccination were shown previously, not only after COVID-19 vaccination [30], but also after other intramuscular vaccines, such as against HPV or pneumococcus [31,32].

## 5. Conclusions

The results of our study show a high reliability of the SARS-CoV-2-specific antibodies as indicators of the infection after week 2 post infection. The positivity in IgG antibody levels is sustained later on, after 9 and 12 months, and significantly correlates to the VNT levels. These findings suggest that serological methods have a valuable place as diagnostics of SARS-CoV-2 infection and its progression, especially after the relatively short time period when direct diagnostic methods can show the presence of the virus. Thus, in the presence of suspicious symptomatology with an absence of positive virus detection results (e.g., swabs for PCR or Ag detection performed late or incorrectly), the serology can serve as the tool for confirming the diagnosis. In addition, the increase in antibody levels after vaccination, confirms that the serology also represents a rapid and easily available tool for the control of vaccination efficiency.

## Figures and Tables

**Table 1:** Basic demographic data.

	Patients	Male	Age Median	Deceased	Female	Age Median	Deceased
Cohort A	225	138 (61.33%)	69	19	87 (38.67%)	70	8
Cohort B	419	285 (68.01%)	42	0	134 (31.99%)	41	0

## Data Availability

Data are available on request due to ethical restrictions.

## Supplementary Materials

The following are available online at https://www.mdpi.com/article/10.3390/v14030526/s1, Table S1: Summary of the analysis of the individual antibody classes in patient cohorts; Table S2: Comparison of serological results in vaccinated and non-vaccinated populations without and with the serologically confirmed reinfection; Table S3: Summary of the virus neutralization test results at the individual time points in both cohorts.
