# Supplementary material for: Long-Term Antibody Response and Vaccination Efficacy in Patients with COVID-19: A Single Center One-Year Prospective Study from the Czech Republic
Source: Viruses. 2022 Mar 4;14(3):526. doi: 10.3390/v14030526 (PMC8949942; doi:10.3390/v14030526)
Supplement: Supplementary file 1 [file viruses-14-00526-s001.zip › viruses-1513279-supplementary.pdf]

**Supplementary Table S1**. Summary of the analysis of the individual antibody classes in patient cohorts over time. The statistical evaluation compares the data from Cohort A and Cohort B. The normality was tested using the Shapiro–Wilk test. Normally distributed data were analyzed using non-parametric t-test. Non-normally distributed data were analyzed by Kruskal–Wallis test with post hoc Mann–Whitney test. The differences were considered significant when $p \leq 0.05$.

| | | IgA | | | IgM | | | IgG | | |
|---|---|---|---|---|---|---|---|---|---|---|
| | | Cohort A | Cohort B | p value | Cohort A | Cohort B | p value | Cohort A | Cohort B | p value |
| Admission | Positive* | 52.17 | NA | | 37.2 | NA | | 28,99 | NA | |
| | Borderline* | 7.24 | NA | | 5.31 | NA | | 6.76 | NA | |
| | Negative* | 40.59 | NA | NA | 57.49 | NA | NA | 64.25 | NA | NA |
| | Pos avg | **6.52** | **NA** | | **4.38** | **NA** | | **4.93** | **NA** | |
| | Pos med | **5.59** | **NA** | | **3.41** | **NA** | | **4.02** | **NA** | |
| Week 2 | Positive* | 98.75 | NA | | 77.75 | NA | | 95.00 | 59.12 | |
| | Borderline* | 0.00 | NA | | 5.00 | NA | | 2.50 | 13.14 | |
| | Negative* | 1.25 | NA | NA | 17.25 | NA | NA | 2.50 | 27.74 | <0.0001 |
| | Pos avg | **9.98** | **NA** | | **5.07** | **NA** | | **7.34** | **3.09** | |
| | Pos med | **10.95** | **NA** | | **4.11** | **NA** | | **7.55** | **2.69** | |
| Month 3 | Positive* | 90.52 | 69.16 | | 14.66 | 2.80 | | 94.83 | 86.84 | |
| | Borderline* | 5.17 | 11.21 | | 6.90 | 3.74 | | 0.86 | 2.63 | |
| | Negative* | 4.31 | 19.63 | <0.0001 | 78.44 | 93.46 | 0.0028 | 4.31 | 10.53 | <0.0001 |
| | Pos avg | **6.74** | **2.70** | | **2.10** | **2.84** | | **6.75** | **3.96** | |
| | Pos med | **5.68** | **2.00** | | **1.85** | **2.92** | | **7.07** | **6.67** | |
| Month 6 | Positive* | 97.62 | 86.30 | | 7.14 | 0.00 | | 98.81 | 82.19 | |
| | Borderline* | 2.38 | 4.11 | | 5.95 | 5.48 | | 0.00 | 4.11 | |
| | Negative* | 0.00 | 9.59 | <0.0001 | 86.91 | 94.52 | 0.0033 | 1.19 | 13.70 | <0.0001 |
| | Pos avg | **9.31** | **5.89** | | **1.97** | **0.00** | | **8.27** | **6.42** | |
| | Pos med | **10.21** | **5.54** | | **2.11** | **0.00** | | **9.41** | **7.99** | |
| Month 9 | Positive* | 92.31 | 78.26 | | 5.13 | 2.42 | | 97.44 | 76.61 | |
| | Borderline* | 7.69 | 5.65 | | 2.56 | 3.23 | | 0.00 | 9.68 | |
| | Negative* | 0.00 | 16.09 | <0.0001 | 92.31 | 94.35 | 0.2152 | 2.56 | 13.71 | <0.0001 |
| | Pos avg | **8.80** | **5.68** | | **1.55** | **1.91** | | **7.88** | **5.26** | |
| | Pos med | **9.96** | **4.17** | | **1.55** | **2.10** | | **9.94** | **4.41** | |
| Month 12 | Positive* | 96.70 | 88.44 | | 5.49 | 0.70 | | 96.70 | 88.44 | |
| | Borderline* | 1.10 | 4.76 | | 0.00 | 0.70 | | 1.10 | 3.08 | |
| | Negative* | 2.20 | 6.80 | 0.095 | 94.51 | 98.60 | 0.1098 | 2.20 | 8.48 | <0.0001 |
| | Pos avg | **8.35** | **7.94** | | **1.68** | **2.73** | | **8.52** | **7.01** | |
| | Pos med | **9.88** | **7.92** | | **1.13** | **2.73** | | **9.30** | **8.40** | |

Notes: * percentage of patients; Cohort A - hospitalized patients; Cohort B - non hospitalized patients; NA - not applicable; Pos avg – average of positive results; Pos med – median value of positive results

**Supplementary Table S2a**. Summary of the analysis of the individual antibody classes in patient cohorts affected by the vaccination or re-infection. The statistical evaluation compares the data from non-vaccinated patients to samples from vaccinated population in both Cohort A and Cohort B. The normality was tested using the Shapiro–Wilk test. Normally distributed data were analyzed using non-parametric t-test. Non-normally distributed data were analyzed by Kruskal–Wallis test with post hoc Mann–Whitney test. The differences were considered significant when p ≤ 0.05.

| | | NON - VACCINATED/NON-REINFECTED | | | | | | VACCINATED | | | | | | | | | | | |
|---|---|---|---|---|---|---|---|---|---|---|---|---|---|---|---|---|---|---|---|
| | | IgA | | IgM | | IgG | | IgA | | | | IgM | | | | IgG | | | |
| | | Coh A | Coh B | Coh A | Coh B | Coh A | Coh B | Coh A | *p value* | Coh B | *p value* | Coh A | *p value* | Coh B | *p value* | Coh A | *p value* | Coh B | *p value* |
| Month 6 | positive* | 91.67 | 76.32 | 12.50 | 0.00 | 95.83 | 71.05 | 100.00 | | 97.06 | | 5.17 | | 0.00 | | 100.00 | | 97.06 | |
| | borderline* | 8.33 | 7.89 | 4.17 | 2.63 | 0.00 | 5.26 | 0.00 | | 0.00 | | 6.90 | | 8.82 | | 0.00 | | 0.00 | |
| | negative* | 0.00 | 15.79 | 83.33 | 97.37 | 4.17 | 23.69 | 0.00 | <0.0001 | 2.94 | <0.0001 | 87.93 | | 91.18 | | 0.00 | <0.0001 | 2.94 | <0.0001 |
| | Pos avg | 5.14 | 3.65 | 2.07 | 0.00 | 5.29 | 3.79 | 10.87 | | 7.83 | | 1.86 | | 0.00 | | 9.35 | | 8.44 | |
| | Pos med | 4.45 | 1.73 | 2.09 | 0.00 | 5.49 | 2.41 | 11.32 | | 7.41 | | 2.13 | | 0.00 | | 9.66 | | 8.98 | |
| Month 9 | positive* | 87.5 | 67.86 | 0.00 | 3.57 | 100.00 | 65.48 | 95.65 | | 96.97 | | 8.70 | | 0.00 | | 95.65 | | 96.97 | |
| | borderline* | 12.5 | 8.33 | 0.00 | 3.57 | 0.00 | 14.29 | 0.00 | | 0.00 | | 4.35 | | 0.00 | | 0.00 | | 0.00 | |
| | negative* | 0.00 | 23.81 | 100.00 | 92.86 | 0.00 | 20.23 | 4.35 | 0.0131 | 3.03 | <0.0001 | 86.95 | | 100.00 | 0.0012 | 4.35 | 0.0026 | 3.03 | <0.0001 |
| | Pos avg | 7.07 | 3.70 | 0.00 | 1.91 | 5.45 | 3.21 | 9.90 | | 8.94 | | 1.54 | | 0.00 | | 9.65 | | 9.02 | |
| | Pos med | 6.10 | 2.98 | 0.00 | 2.10 | 4.03 | 2.86 | 12.40 | | 8.89 | | 1.54 | | 0.00 | | 10.41 | | 9.36 | |
| Month 12 | positive* | 87.5 | 74.00 | 0.00 | 2.00 | 87.5 | 66.00 | 100.00 | | 96.74 | | 5.41 | | 0.00 | | 100.00 | | 100.00 | |
| | borderline* | 6.25 | 12.00 | 6.25 | 0.00 | 6.25 | 8.00 | 0.00 | | 0.00 | | 0.00 | | 1.10 | | 0.00 | | 0.00 | |
| | negative* | 6.25 | 14.00 | 93.75 | 98.00 | 6.25 | 26.00 | 0.00 | <0.0001 | 3.26 | <0.0001 | 94.59 | | 98.90 | | 0.00 | 0.0002 | 0.00 | <0.0001 |
| | Pos avg | 4.94 | 4.13 | 0.00 | 2.73 | 5.38 | 3.15 | 8.98 | | 9.30 | | 1.84 | | 0.00 | | 9.09 | | 8.45 | |
| | Pos med | 3.39 | 3.37 | 0.00 | 2.73 | 3.71 | 2.38 | 9.99 | | 9.92 | | 1.68 | | 0.00 | | 9.44 | | 9.16 | |

Notes: * percentage of patients; Coh A – Cohort A (hospitalized patients); Coh B – Cohort B (non hospitalized patients); NA - not applicable; Pos avg – average of positive results; Pos med – median value of positive results

**Supplementary Table S2b**. Summary of the analysis of the individual antibody classes in patient cohorts affected by the vaccination. The statistical evaluation compares the data from patients vaccinated ≤ 4 months with data of those vaccinated ≥ 5 months in both Cohort A and Cohort B The normality was tested using the Shapiro–Wilk test. Normally distributed data were analyzed using non-parametric t-test. Non-normally distributed data were analyzed by Kruskal–Wallis test with post hoc Mann–Whitney test. The differences were considered significant when p ≤ 0.05.

| | | VACCINATED <=4M | | | | | | VACCINATED >=5 M | | | | | | | | | | | |
|---|---|---|---|---|---|---|---|---|---|---|---|---|---|---|---|---|---|---|---|
| | | IgA | | IgM | | IgG | | IgA | | | | IgM | | | | IgG | | | |
| | | Coh A | Coh B | Coh A | Coh B | Coh A | Coh B | Coh A | *p value* | Coh B | *p value* | Coh A | *p value* | Coh B | *p value* | Coh A | *p value* | Coh B | *p value* |
| **Month 9** | positive* | 100.00 | 100.00 | 11.11 | 0.00 | 100.00 | 100.00 | 80.00 | | 90,91 | | 0.00 | | 0.00 | | 80.00 | | 90.91 | |
| | borderline* | 0.00 | 0.00 | 5.56 | 0.00 | 0.00 | 0.00 | 20.00 | | 0.00 | | 0.00 | | 0.00 | | 0.00 | | 0.00 | |
| | negative* | 0.00 | 0.00 | 83.33 | 100.00 | 0.00 | 0.00 | 0.00 | 0.2032 | 9,09 | 0.0033 | 100.00 | 0.3530 | 100.00 | 0.4446 | 20.00 | 0.0089 | 9.09 | 0.0042 |
| | Pos avg | 10.10 | 10.21 | 1.54 | 0.00 | 10.01 | 9.44 | 7.66 | | 6.15 | | 0.00 | | 0.00 | | 8.00 | | 8.11 | |
| | Pos med | 12.73 | 11.84 | 1.54 | 0.00 | 10.58 | 9.59 | 6.72 | | 6.02 | | 0.00 | | 0.00 | | 9.83 | | 8.58 | |
| **Month 12** | positive* | 100.00 | 100.00 | 7.41 | 0.00 | 100.00 | 100.00 | 97.87 | | 93.18 | | 4.44 | | 0.00 | | 97.87 | | 100.00 | |
| | borderline* | 0.00 | 0.00 | 0.00 | 2.08 | 0.00 | 0.00 | 0.00 | | 0.00 | | 0.00 | | 0.00 | | 0.00 | | 0.00 | |
| | negative* | 0.00 | 0.00 | 92.59 | 97.92 | 0.00 | 0.00 | 2.23 | 0.0009 | 6.82 | < 0.0001 | 95.56 | 0.9163 | 100.00 | 0.2321 | 2.23 | 0.0399 | 0.00 | 0.0061 |
| | Pos avg | 9.59 | 10.96 | 2.00 | 0.00 | 9.23 | 9.03 | 8.62 | | 7.36 | | 1.68 | | 0.00 | | 9.01 | | 7.81 | |
| | Pos med | 11.46 | 11.58 | 2.00 | 0.00 | 7.72 | 9.43 | 9.92 | | 7.23 | | 1.68 | | 0.00 | | 9.20 | | 8.40 | |

Notes: * percentage of patients; Coh A – Cohort A (hospitalized patients); Coh B – Cohort B (non hospitalized patients); Pos avg – average of positive results; Pos med – median value of positive results

**Supplementary Table S2c**. Summary of the analysis of the individual antibody classes in patient cohorts affected by re-infection. The table shows the data from non-vaccinated patients to samples from non-vaccinated patients with serologicaly marks of re-infection in both Cohort A and Cohort. The statistical analysis was not performed for low sample numbers.

| | | NON - VACCINATED/NON-REINFECTED | | | | | | NON-VACCINATE/REINFECTED | | | | | |
|---|---|---|---|---|---|---|---|---|---|---|---|---|---|
| | | IgA | | IgM | | IgG | | IgA | | IgM | | IgG | |
| | | Coh A | Coh B | Coh A | Coh B | Coh A | Coh B | Coh A | Coh B | Coh A | Coh B | Coh A | Coh B |
| Month 6 | positive* | 91.67 | 76.32 | 12.50 | 0.00 | 95.83 | 71.05 | 100.00 | 100.00 | 0.00 | 0.00 | 100.00 | 100.00 |
| | borderline* | 8.33 | 7.89 | 4.17 | 2.63 | 0.00 | 5.26 | 0.00 | 0.00 | 0.00 | 0.00 | 0.00 | 0.00 |
| | negative* | 0.00 | 15.79 | 83.33 | 97.37 | 4.17 | 23.69 | 0.00 | 0.00 | 100.00 | 100.00 | 0.00 | 0.00 |
| | Pos avg | 5.14 | 3.65 | 2.07 | 0.00 | 5.29 | 3.79 | 9.37 | 6.59 | 0.00 | 0.00 | 8.45 | 5.16 |
| | Pos med | 4.45 | 1.73 | 2.09 | 0.00 | 5.49 | 2.41 | 9.37 | 6.59 | 0.00 | 0.00 | 8.45 | 5.16 |
| Month 9 | positive* | 87.5 | 67.86 | 0.00 | 3.57 | 100.00 | 65.48 | NA | 100.00 | NA | 0.00 | NA | 100.00 |
| | borderline* | 12.5 | 8.33 | 0.00 | 3.57 | 0.00 | 14.29 | NA | 0.00 | NA | 0.00 | NA | 0.00 |
| | negative* | 0.00 | 23.81 | 100.00 | 92.86 | 0.00 | 20.23 | NA | 0.00 | NA | 100.00 | NA | 0.00 |
| | Pos avg | 7.07 | 3.70 | 0.00 | 1.91 | 5.45 | 3.21 | NA | 7.53 | NA | 0.00 | NA | 4.84 |
| | Pos med | 6.10 | 2.98 | 0.00 | 2.10 | 4.03 | 2.86 | NA | 8.80 | NA | 0.00 | NA | 5.26 |
| Month 12 | positive* | 87.5 | 74.00 | 0.00 | 2.00 | 87.5 | 66.00 | 100.00 | 100.00 | 0.00 | 0.00 | 100.00 | 100.00 |
| | borderline* | 6.25 | 12.00 | 6.25 | 0.00 | 6.25 | 8.00 | 0.00 | 0.00 | 0.00 | 0.00 | 0.00 | 0.00 |
| | negative* | 6.25 | 14.00 | 93.75 | 98.00 | 6.25 | 26.00 | 0.00 | 0.00 | 100.00 | 100.00 | 0.00 | 0.00 |
| | Pos avg | 4.94 | 4.13 | 0.00 | 2.73 | 5.38 | 3.15 | 10.62 | 10.37 | 0.00 | 0.00 | 10.81 | 6.04 |
| | Pos med | 3.39 | 3.37 | 0.00 | 2.73 | 3.71 | 2.38 | 10.62 | 11.78 | 0.00 | 0.00 | 10.80 | 7.10 |

Notes: * percentage of patients; Coh A – Cohort A (hospitalized patients); Coh B – Cohort B (non hospitalized patients); NA - not applicable; Pos avg – average of positive results; Pos med – median value of positive results

**Supplementary Table S3**. Summary of the virus neutralization test results at the individual time points in both cohorts. The numbers represents a proportion of samples in percents for with the indicated VNT levels.

| Virus neutralization titer | Cohort A (%) | Cohort B (%) | |
|---|---|---|---|
| **<40** | 56.10 | NA | **Admission** |
| **40** | 11.70 | NA | |
| **80** | 10.73 | NA | |
| **160-320** | 12.68 | NA | |
| **>640** | 8.79 | NA | |
| **<40** | 2.44 | 23.08 | **Week 2** |
| **40** | 8.54 | 18.46 | |
| **80** | 4.88 | 43.08 | |
| **160-320** | 51.22 | 12.31 | |
| **>640** | 32.92 | 3.07 | |
| **<40** | 6.36 | 28.16 | **Month 3** |
| **40** | 12.73 | 34.48 | |
| **80** | 18.18 | 21.26 | |
| **160-320** | 39.09 | 14.37 | |
| **>640** | 23.64 | 1.73 | |
| **<40** | 3.77 | 0.00 | **Month 6** |
| **40** | 1.89 | 0.00 | |
| **80** | 5.66 | 66.67 | |
| **160-320** | 9.43 | 33.33 | |
| **>640** | 79.25 | 0.00 | |
| **<40** | 0.00 | 36.90 | **Month 9** |
| **40** | 30.00 | 32.14 | |
| **80** | 40.00 | 14.29 | |
| **160-320** | 30.00 | 15.48 | |
| **>640** | 0.00 | 1.19 | |
| **<40** | 0.00 | 27.27 | **Month 12** |
| **40** | 14.29 | 9.09 | |
| **80** | 7.14 | 12.73 | |
| **160-320** | 14.29 | 18.18 | |
| **>640** | 64.29 | 32.73 | |
